# Managing occupational health among goldminers in Ghana: Modelling the likelihood of experiencing occupational related health problems

**DOI:** 10.1371/journal.pone.0254449

**Published:** 2021-07-15

**Authors:** Simon Appah Aram

**Affiliations:** 1 Research Center for Smart Mine and Intelligent Equipment, Taiyuan University of Technology, Taiyuan, People’s Republic of China; 2 College of Safety and Emergency Management Engineering, Taiyuan University of Technology, Taiyuan, People’s Republic of China; Al Mansour University College-Baghdad-Iraq, IRAQ

## Abstract

**Background:**

The importance of goldmining to Ghana’s development cannot be overestimated. However, the associated morbidities and mortalities resulting from occupational exposure to health hazards and the general cost associated with it is critical. In managing occupational health, a simple comprehension of the main determinants of the problem is required.

**Methods:**

A cross-sectional survey of 504 goldminers was fitted to a nested binary logistic regression model to evaluate the independent effect of subsector departments, compositional attributes, contextual factors and working conditions on goldminers’ likelihood of experiencing occupational related health problems.

**Results:**

Subsector department was robust and persisted in predicting experiencing occupational related health problems in all three models. Goldminers who were in artisanal small scale (ASM) non-production, large scale (LSM) production and LSM non-production were less likely to experience occupational related health problems as compared to their counterparts in ASM production. For the compositional factors, female goldminers were more likely to experience occupational related health problems as compared to their male co-workers. Goldminers who were married and also older miners were more likely to experience occupational related health problems as compared to the unmarried and the relatively younger goldminers. At the contextual level, miners who worked on shift-based schedule and also miners who lived close to mine sites were more likely to experience occupational related health problems. Among the working conditions, goldminers who worked in good health conditions were less likely to experience occupational related health problems. Surprisingly, goldminers who rated their safety conditions as good were more likely to experience occupational related health problems.

**Conclusion:**

Gold miners are exposed to different health risk scenarios across subsectors and departments. These conditions need critical attention and action from industry stakeholders. Programs that promote self-care culture should be promoted, especially in the ASM subsector. PPE’s could be relied on for protection in the mining industry but they should be the last line of defense and not to replace preventive measures and actions.

## Introduction

Globally, mining has been going on since prehistoric time. Mining worldwide is improving and facilitating the development of economies worldwide [1]. However, goldmining whether large scale (LSM) or small scale (ASM) still remains one of the most problematic, dirty and dangerous occupations worldwide [2]. The mining industry are among the top three sectors with the highest morbidities and mortalities in the world [3]. Hence, the health and safety culture in mining demands serious attention and action.

Mine workers engage in complex work processes, which may expose them to various types and levels of health risks and hazards [4]. According to the International Labor Organization (ILO) [5], an estimated 2.34 million people die every twelve months from work-related accidents and diseases including mine workers. Of these, the vast majority, 2.02 million people die from work-related diseases. Also, out of the estimated 6,300 work related deaths that happen every 24 hours globally, 5500 are caused by various types of work-related diseases. The ILO further estimates that 160 million cases of non-fatal work-related diseases and 264 million non-fatal accidents, occur annually leading to approximately three days of absence from work. Mortality rate rankings by industrial divisions have consistently indicated that workers in mining and construction have had higher relative risks of work-related fatalities than those in manufacturing, trade, and service industries [4].

Despite the enormous economic benefits associated with mining, its detrimental effects on the health of miners cannot be overlooked. Mining activities, both surface and underground, comes along with numerous health externalities [6,7]. Experiencing occupational related ill-health has huge social and economic implications for individuals, their families and their communities [8]. They also have economic impacts in the form of direct and indirect costs to society as a whole. Total costs of occupational accidents and diseases have been estimated to be between 1% and 3% of GDP in various countries [8] and the ILO [5] estimates that 4% of global GDP is lost due to occupational accidents and diseases. Occupational ill-health could lead to disability, reliance on benefits (if they exist), early retirement, the loss of a breadwinner and poverty.

It is hypothesized that this could even be more in limited resource settings, where mining regulations are less enforced and, the health and safety of miners is of less concern. ASM in particular suffers the most due to lack of investment and regulations which is not the case in the LSM, where attention from stakeholders and investment levels are increasing. It is estimated that the working conditions of ASM workers are worse than in the LSM subsector with the former characterized by heavy manual work and no facilities for safety and health. Generally, improvement in working conditions in the mines has been recommended to lessen the burden of diseases and to address the far reaching negative health effects on the mine worker.

The associated morbidities and mortalities resulting from occupational exposure to health hazards and the general cost associated with it in Ghana is crucial. In managing occupational health, a simple comprehension of the main determinants of the problem is required. In this study, the independent effects of the subsector department, compositional attributes, contextual factors and working conditions on goldminers’ likelihood of experiencing occupational related health problems was evaluated using a nested binary logistic regression model.

## Methods

### Study area

The study was conducted in the Southwestern part of Ghana. This area is part of the Birimian and Tarkwain formations [9]. The existence of high mineral deposits in this region is as a result of the Birimian and Tarkwain geological formations. The area’s geology makes it extremely appealing to gold mining and it is home to some of the biggest large scale mining companies and also has numerous artisanal mining concessions. The area provides outstanding opportunities to better comprehend the nature and risks involved in goldmining in Ghana. This is because the area has the highest per capita concentrations of goldminers in Ghana.

### Data collection and sampling procedure

This study is part of a research project that assessed the working conditions of goldminers in Ghana. Data collection took place from January 2018 to December 2019. The questionnaire was adapted and developed from other related studies, reviewed and accepted by the University of Cape Coast institutional review board in Ghana. The questionnaire had three parts: contextual aspects, compositional characteristics and working condition quality measures. It comprised of closed-ended questions which provided a variety of multiple-choice answers from which the respondents were given the opportunity to tick as applicable. To ensure its feasibility and content validity, the questionnaire was tested among 15 participants from Boboobo, a town with similar socioeconomic background to the respondents of the study. The pilot group was first asked to complete the questionnaire, and comment on the comprehensibility of the questions; this led to minor modifications of the questionnaire to improve understanding. Participants were randomly recruited for the survey. Participants who had worked for less than a month and participants less than 18 years old were not recruited. Overall, 504 out of the 510 recruited goldminers agreed to participate in the survey. Reasons such as time constraints were cited for the inability to participate in the study. The sample size was estimated based on a 95% confidence interval, 50% estimated population proportion and at a 5% error rate.

### Derivation of response variable

The response variable for this study was experiencing occupational related health problems by goldminers in Ghana. Respondents were asked if they often experience symptoms of any of the goldmining related diseases since they started working in the mining industry. If the respondent ticked a minimum of one disease, it was indicated as “Yes” and if none was ticked it was indicated as “No”. The dichotomous response was coded as 0 (for no) and 1 (for yes).

### Derivation of independent variables

#### Key predictor variables

The key predictor for this study was derived from combining two variables; subsector and department. This produced the predictor called subsector department with four mutually exclusive groups; ASM production, ASM non-production, LSM production and LSM non-production.

#### Compositional and contextual factors

In this study compositional factors referred to socio-demographic characteristics of the goldminers. These factors included age, sex, marital status, education and years of experience. The contextual factors were medical checkup, years of residence, shift regime and proximity to mine sites.

#### Working conditions

Participants were asked to rate their working conditions. Each of the four indicators, with a series of questions, was evaluated as very poor (1), poor (2), good (3), very good (4), and excellent (5)”. Total scores that were greater than 3 were considered as “good” and scores less than 3 were considered as “poor”. For this study, “Health” referred to the functional status of gold miners. These include emotional wellbeing, physical fitness and rate of change in health status. “Safety" denotes the availability of appropriate personal protective equipment such as protective clothing, goggles, gloves, and institutionalization of practices such as safe disposal of hazardous materials, allowed levels of noise and protection against fall. “Environment” is generally described to encompass both physical and social scopes. These include but not limited to resources required for the job, perceptions of their workspace quality and setting, the physical surroundings and space availability. “Economic conditions” also include wages or income, employment benefits, incentives and workload that cumulatively influence the productivity of goldminers [10,11].

The selection of these variables as key predictors, compositional and contextual factors, and working conditions were based on literature, practical significance, theoretical relevance and parsimony.

### Data analyses

The ensuing analytical procedures were followed. Firstly, descriptive analysis was conducted to determine the percentages and distributions of the characteristics of goldminers. After that, Pearson’s chi-square statistic was used to test and describe the relationship between the categorical independent variables and experiencing occupational related health problems by goldminers. A negative log-log bivariate regression was carried out to ascertain the “one-on-one” predictive relationship of the predictors and the dependent variable before the multivariate model was implemented. The data was subjected to multivariate statistical analysis to examine the relationships and proportions between factors that influence experiencing occupational related health problems while controlling for theoretically relevant compositional, contextual and working condition factors using a nested binary logistic regression model.

Logistic regression allows the model to be related to the response variable via a link function and by allowing the magnitude of the variance of each measurement to be a function of its predicted value under the assumption of a binary response (Yes/No). Via the link function, there are several potential alternatives: the logit model, probit model, negative log-log and complementary log-log models. Both logit and probit link functions have the same property which is the probability that an observation in a specified category of a binary outcome variable (experiencing occupational related health problems or not) has the same probability of approaching 0 as well as approaching 1 (50% No, 50% Yes). If the observations of a binary outcome have an asymmetrical success of probability, that is, fewer 0s than 1s or more 0s than 1s, then the link function complementary log-log or negative log-log is chosen respectively. In this study, 68.06% of the goldminers did not experience occupational related health problems indicating that the probability of the outcome was asymmetrical. For this reason, the negative log-log link function was appropriate for modelling the dependent variable.

The odds ratios (OR) were built in a nested model starting from key predictor and compositional factors model, contextual factors model and working conditions model. An OR of 1 meant that higher values of the predictor did not affect the odds of experiencing occupational related health problems; OR >1 meant that the predictor was associated with higher odds of experiencing occupational related health problems; and OR<1 meant the predictor variable was associated with lower odds of experiencing occupational related health problems. All statistical analyses were performed using Stata 15 (StataCorp, College Station, Texas) SE software at a statistical significance of 0.05 and at a confidence interval of 95%.

### Ethical statement

Ethical approval was sought from the Ghana Health Service Ethical Review Board to conduct the study. The purpose of the study and other details were disclosed to the authorities and participants. Oral consent was sought from participants before the study started as required by the minerals commission of Ghana. Participants were not financially induced or coerced to take part in the study. It was explained to them that their participation was voluntary. They were also informed that the information provided will contribute to the improvement in their working conditions in Ghana.

## Results

### Descriptive and inferential results

Table 1 presents the descriptive and inferential results of the study. Participants were between 18 to 60 years. Years of experience in the mines was between 1 to 52 years (M = 7.092, SD = 6.48256). Notably across subsectors and departments, 56.18% of ASM miners who worked in production departments did not experience occupational related health challenges while 76.47% of ASM non-production miners did not experience occupational related health problems. For LSM miners in productions, 16.98% experienced occupational related health challenges while only 15.89% LSM miners in non-production experienced occupational related health problems. Among miners who reported poor health conditions at work, 58.43% experienced occupational related health challenges while only 26.27% of miners who reported good health conditions at work experienced occupational related health challenges. Goldminers who reported poor safety, environmental and economic conditions had 38.96%, 40.19% and 39.85% respectively indicating they experienced occupational related health challenges. Additionally, goldminers who reported good safety, environmental and economic conditions had 25.10%, 26.10% and 22.75% respectively indicating they experienced occupational related health challenges.

**Table 1:** Demographic characteristics and percentage distribution of experiencing occupational related health problems by predictor variables.

Variables	Weighed Frequency	Weighed Percentage	Experiencing occupational related health problems		Inferential statistics
			No (%)	Yes (%)	
**Subsector + Department** **χ^2^ (3) = 42.2523, p<0.001**					
ASM Production	283	56.15	159 (56.18)	124 (43.82)	
ASM Non-production	17	3.37	13 (76.47)	4 (23.53)	
LSM Production	53	10.52	44 (83.02)	9 (16.98)	
LSM Non-production	151	29.96	127 (84.11)	24 (15.89)	
**Age χ^2^ (3) = 11.0799, p<0.05**					
18–24 years	167	33.13	126(75.45)	41 (24.55)	
25–34 years	242	48.02	154 (63.64)	88 (36.36)	
35–54 years	67	13.29	40 (59.70)	27 (40.30)	
Above 55 years	28	5.56	23 (82.14)	5 (17.86)	
**Gender χ^2^ (1) = 7.0899, p<0.05**					
Male	434	86.11	305 (70.28)	129 (29.72)	
Female	70	13.89	38 (54.29)	32 (45.71)	
**Marital status χ^2^ (1) = 10.0570, p<0.05**					
Single	334	66.27	243 (72.75)	91 (27.25)	
Married	170	33.73	100 (58.82)	70 (41.18)	
**Education χ^2^ (2) = 7.5604, p<0.05**					
No formal/Primary/Junior High	202	40.08	129 (63.86)	73 (36.14)	
Senior High	131	25.99	84 (64.12)	47 (35.88)	
Tertiary	171	33.93	130 (76.02)	41 (23.98)	
**Experience χ^2^ (2) = 2.8703, p = 0.238**					
1–5 years	289	57.34	192 (66.44)	97 (33.56)	
6–10 years	118	23.41	78 (66.10)	40 (33.90)	
Above 10 years	97	19.25	73 (75.26)	24 (24.74)	
**Medical Checkup χ^2^ (1) = 4.9811, p<0.05**					
No	196	38.89	122 (62.24)	74 (37.76)	
Yes	308	61.11	221 (71.75)	87 (28.25)	
**Years of Residence χ^2^ (1) = 4.4291, p = 0.109**					
1–5 years	146	28.97	97 (66.44)	49 (33.56)	
6–10 years	132	26.19	82 (62.12)	50 (37.88)	
Above 10 years	226	44.84	164 (72.57)	62 (27.43)	
**Shift χ^2^ (1) = 1.4126, p = 0.235**					
No	392	78.4	263 (66.75)	131 (33.25)	
Yes	108	21.6	80 (72.73)	30 (27.27)	
**Proximity χ^2^ (1) = 18.7400, p<0.001**					
No	268	53.17	205 (76.49)	63 (23.51)	
Yes	236	46.83	138 (58.47)	98 (41.53)	
**Health conditions χ^2^ (1) = 34.8684, p<0.001**					
Poor	89	17.66	37 (41.57)	52 (58.43)	
Good	415	82.34	306 (73.73)	109 (26.27)	
**Safety conditions χ^2^ (1) = 11.1285, p<0.05**					
Poor	249	49.4	152 (61.04)	97 (38.96)	
Good	255	50.6	191 (74.90)	64 (25.10)	
**Environmental conditions χ^2^ (1) = 11.1707, p<0.05**					
Poor	209	41.47	125 (59.81)	84 (40.19)	
Good	295	58.53	218 (73.90)	77 (26.10)	
**Economic conditions χ^2^ (1) = 16.8622, p<0.001**					
Poor	271	53.77	163 (60.15)	108 (39.85)	
Good	233	46.23	180 (77.25)	53 (22.75)	

Table 1 also presents Pearson’s chi-square test of independence. Pearson’s chi-square test was used to determine whether the observed differences in experiencing occupational related health problems and compositional factors as well as contextual and working conditions factors were independent. For the key predictor, there was statistically significant association between subsector department (χ^2^ (3) = 42.2523, p<0.001) and experiencing occupational related health challenges. For the compositional variables, age (χ^2^ (3) = 11.0799, p<0.05), gender (χ^2^ (1) = 7.0899, p<0.05), marital status (χ^2^ (1) = 10.0570, p<0.05) and education (χ^2^ (2) = 7.5604, p<0.001) had statistically significant association with experiencing occupational related health problems. For the contextual factors, medical checkup (χ^2^ (1) = 4.9811, p<0.05) and proximity (χ^2^ (1) = 18.7400, p<0.001) had statistically significant associations with experiencing occupational related health challenges. There was however no relationship between experience (χ^2^ (2) = 2.8703, p = 0.238), years of residence (χ^2^ (1) = 4.4291, p = 0.109), shift (χ^2^ (1) = 1.4126, p = 0.235) and experiencing occupational related health problems. With working conditions, health conditions (χ ^2^ (1) = 34.8684, p<0.001), safety conditions (χ^2^ (1) = 11.1285, p<0.005), environmental conditions (χ^2^ (1) = 11.1707, p<0.005) and economic conditions (χ^2^ (1) = 16.8622, p<0.001) had statistically significant relationships with experiencing occupational related health problems.

### Bivariate logistic regression of experiencing occupational related health problems and predictor variables

For the key predictors in the bivariate analysis in Table 2, miners in LSM production and LSM non-production departments were 54% and 55% less likely to experience occupational related health problems as compared to their counterparts in ASM production department.

**Table 2:** Bivariate negative log-log regression of experiencing occupational related health problems by goldminers.

Variables	OR	Robust SE	p-value	Conf. Interval	
**Subsector+ Department (ref: ASM Production)**					
ASM Non-production	0.570	0.179	0.073	0.309	1.054
LSM Production	0.465	0.088	**0.000**	0.321	0.675
LSM Non-production	0.449	0.059	**0.000**	0.347	0.579
**Age (ref: 18–24 years)**					
25–34 years	1.388	0.178	**0.010**	1.080	1.785
35–54 years	1.545	0.294	**0.022**	1.064	2.243
Above 55 years	0.815	0.208	0.422	0.495	1.343
**Gender (ref: Male)**					
Female	1.550	0.275	**0.013**	1.095	2.194
**Marital status (ref: Single)**					
Married	1.465	0.182	**0.002**	1.149	1.869
**Education (ref: No formal/Primary/Junior High)**					
Senior High	0.993	0.145	0.961	0.745	1.323
Tertiary	0.713	0.094	**0.011**	0.550	0.924
**Experience (: 1–5 years)**					
6–10 years	1.009	0.142	0.948	0.765	1.331
Above 10 years	0.782	0.116	0.096	0.585	1.044
**Medical Checkup (ref: No)**					
Yes	0.770	0.091	**0.028**	0.611	0.972
**Years of Residence (ref: 1–5 years)**					
6–10 years	1.125	0.176	0.454	0.827	1.529
Above 10 years	0.844	0.115	0.212	0.647	1.101
**Shift (ref: No)**					
Yes	0.848	0.116	0.225	0.649	1.107
**Proximity (ref: No)**					
Yes	1.647	0.192	**0.000**	1.311	2.069
**Health conditions (ref: Poor)**					
Good	0.402	0.071	**0.000**	0.284	0.569
**Safety conditions (ref: Poor)**					
Good	0.682	0.078	**0.001**	0.544	0.854
**Environmental conditions (ref: Poor)**					
Good	0.679	0.080	**0.001**	0.539	0.855
**Economic conditions (ref: Poor)**					
Good	0.621	0.072	**0.000**	0.496	0.779

Of the compositional factors, female goldminers were 1.55 times more likely to experience occupational related health problems as compared to their male counterparts. Likewise, miners who are married were 1.465 times more likely to experience occupational related health problems than their unmarried mates. Mine workers who were between 25–34 years and 35–54 were 1.388 and 1.454 times respectively more likely to experience occupational related health problems than their 18–24 years counterparts. Also, goldminers who had tertiary education had a 29% chance of not experiencing occupational related health problems as compared to those with no formal or primary or junior high school education. Experience was not a significant predictor of experiencing occupational related health problems.

For the contextual factors, goldminers who routinely go for medical checkup were 23% less likely to experience occupational related health problems than those who did not voluntarily go for checkup. Similarly, goldminers who live close to mine sites were 1.647 times more likely to experience occupational related health problems. Years of residence and shift were not significant predictors of experiencing occupational related health problems by goldminers at the bivariate level.

For working conditions, goldminers who reported good health conditions were 92% less likely to experience occupational related health problems as compared to those who reported poor health conditions. Also, goldminers who reported good safety, environmental and economic conditions were 31%, 32% and 37% respectively less probable to experience occupational related health problems as compared to those who reported poor safety, environmental and economic conditions.

### Multivariate negative log-log regression model predicting experiencing occupational related health problems by gold mine workers

Table 3 is a nested multivariate logistic regression showing the three models; key predictors + compositional model, contextual model and working conditions model for predicting experiencing occupational related health problems by goldminers in Ghana. In model 1 (key predictors + compositional factors), miners in LSM production and LSM non-production departments were 68% and 71% respectively less likely to experience occupational related health problems as compared to their counterparts in ASM production. ASM non-production however was not a significant predictor. For the compositional factors, female miners were 2.102 times more likely to experience occupational related health problems as compared to their male colleagues. Likewise, goldminers who are married were 1.559 times more likely to experience occupational related health problems as compared to the unmarried miners. Goldminers who were between 25–34 years and 35–54 years were 1.785 and 1.813 times respectively more likely to experience occupational related health problems as compared to their counterparts who were between the ages of 18–24 years. However, education and experience were not statistically significant predictors.

**Table 3:** Multivariate negative log-log regression model predicting experiencing occupational related health problems by gold mine workers.

Variables	Model 1: Key predictors + Compositional factors					Model 2: Key predictors + Compositional + Contextual factors					Model 2: Key predictors + Compositional + Contextual + Working conditions				
	OR	Robust SE	p-value	Conf. Interval		OR	Robust SE	p-value	Conf. Interval		OR	Robust SE	p-value	Conf. Interval	
**Subsector + Department (ref: ASM Production)**															
ASM Non-production	0.629	0.226	0.197	0.311	1.272	0.540	0.185	0.072	0.276	1.056	0.269	0.099	**0.000**	0.131	0.555
LSM Production	0.321	0.073	**0.000**	0.206	0.500	0.216	0.060	**0.000**	0.126	0.371	0.094	0.036	**0.000**	0.044	0.200
LSM Non-production	0.294	0.052	**0.000**	0.208	0.417	0.253	0.050	**0.000**	0.171	0.374	0.102	0.036	**0.000**	0.051	0.204
**Age (ref: 18–24 years)**															
25–34 years	1.785	0.280	**0.000**	1.312	2.429	1.692	0.281	**0.002**	1.221	2.344	1.837	0.328	**0.001**	1.294	2.607
35–54 years	1.813	0.466	**0.021**	1.096	3.001	1.799	0.459	**0.021**	1.092	2.966	1.964	0.493	**0.007**	1.201	3.211
Above 55 years	0.797	0.274	0.509	0.406	1.563	0.890	0.308	0.736	0.452	1.753	1.096	0.367	0.784	0.569	2.111
**Gender (ref: Male)**															
Female	2.012	0.390	**0.000**	1.376	2.941	1.652	0.332	**0.012**	1.115	2.449	1.558	0.337	**0.040**	1.020	2.379
**Marital status (ref: Single)**															
Married	1.559	0.251	**0.006**	1.137	2.138	1.706	0.283	**0.001**	1.232	2.363	1.694	0.273	**0.001**	1.235	2.324
**Education (ref: No formal/Primary/Junior High)**															
Senior High	1.226	0.204	0.221	0.885	1.698	1.045	0.181	0.798	0.745	1.467	0.965	0.167	0.834	0.687	1.353
Tertiary	1.149	0.222	0.474	0.786	1.679	1.097	0.218	0.640	0.743	1.620	1.184	0.232	0.388	0.807	1.739
**Experience (ref: 1–5 years)**															
6–10 years	0.992	0.153	0.959	0.733	1.342	0.937	0.158	0.699	0.673	1.304	1.008	0.172	0.963	0.721	1.409
Above 10 years	0.704	0.153	0.107	0.460	1.078	0.693	0.158	0.108	0.443	1.084	0.765	0.172	0.233	0.492	1.188
**Medical Checkup (ref: No)**															
Yes						1.019	0.151	0.901	0.761	1.363	0.869	0.136	0.369	0.640	1.180
**Years of Residence (ref: 1–5 years)**															
6–10 years						1.120	0.197	0.520	0.793	1.581	1.054	0.190	0.771	0.740	1.502
Above 10 years						0.924	0.159	0.646	0.659	1.295	0.933	0.164	0.695	0.662	1.317
**Shift (ref: No)**															
Yes						1.768	0.347	**0.004**	1.203	2.597	1.912	0.375	**0.001**	1.302	2.808
**Proximity (ref: No)**															
Yes						1.744	0.226	**0.000**	1.352	2.248	1.741	0.232	**0.000**	1.340	2.261
**Health conditions (ref: Poor)**															
Good											0.540	0.120	**0.006**	0.348	0.836
**Safety conditions (ref: Poor)**															
Good											3.082	1.090	**0.001**	1.540	6.166
**Environmental conditions (ref: Poor)**															
Good											1.548	0.346	0.051	0.999	2.400
**Economic conditions (ref: Poor)**															
Good											0.737	0.201	0.263	0.432	1.257

In model 2, where contextual factors were accounted for, the key predictors persisted in predicting experiencing occupational related health problems. In this instance, miners in LSM production and LSM non-production departments were 78% and 75% respectively less likely to experience occupational related health problems as compared to their counterparts in ASM production. For the compositional factors, it was evident that gender, marital status and age were persistent in determining the likelihood of experiencing occupational related health problems. Female miners were 1.652 times more likely to experience occupational related health problems as compared to their male colleagues. Likewise, goldminers who are married were 1.706 times more likely to experience occupational related health problems as compared to the unmarried miners. Goldminers who were between 25–34 (OR = 1.692, p<0.05) years and 35–54 years (OR = 1.799, p<0.05) were more likely to experience occupational related health problems as compared to their counterparts who were between the ages of 18–24 years. Education and experience were still not statistically significant predictors. Among the contextual factors, goldminers who worked on a shift-based schedule and also goldminers who stayed close to mine sites were 1.768 and 1.744 times respectively more likely to experience occupational related health problems. Medical checkup and years of residence in the mining community were not statistically significant predictors.

In model 3, where working conditions were controlled for, LSM production and LSM non-production departments were still associated to experiencing occupational related health problems by goldminers in Ghana. A new relationship however appeared, indicating mediation by the working condition factors. In this case, ASM non-production became statistically significant in predicting experiencing occupational related health problems. This clearly indicated that the subsector and the department of the miner were robust in predicting experiencing occupational related health problems.

For the compositional factors, gender, marital status and age were still statistically significant in predicting experiencing occupational related health problems, similar to that of model 1 and 2. In this instance, female miners were 1.558 times more likely to experience occupational related health problems as compared to their male colleagues. Likewise, goldminers who are married were 1.694 times more likely to experience occupational related health problems as compared to the unmarried miners. Goldminers who were between 25–34 (OR = 1.837, p<0.05) years and 35–54 years (OR = 1.964, p<0.05) had higher odds of experiencing occupational related health problems as compared to their counterparts who were between the ages of 18–24 years.

For the contextual factors, shift and proximity were still statistically significant in predicting experiencing occupational related health problems, similar to that of model 2. Goldminers who were on a shift regime and also miners who stayed close to mine sites were 1.912 and 1.741 times respectively more likely to experience occupational related health problems.

At the working conditions level, goldminers who reported good health conditions were 46% less likely to experience occupational related health problems as compared to miners who reported poor health conditions. Contrariwise, goldminers who reported good safety conditions were 3.082 times more probable to experience occupational related health problems as compared to their counterparts who reported poor safety conditions. Environmental and economic conditions were not significant predictors.

## Discussion

In this study, the effect of subsector department on the likelihood of experiencing occupational related health problems was assessed, while controlling for compositional attributes, contextual factors and working conditions of goldminers in Ghana. The importance of goldmining to Ghana’s development cannot be overemphasized. However, the associated morbidities and mortalities resulting from occupational exposure and the general cost associated with it is critical. A key requirement to reducing health and safety risks to minimal levels, is the implementation of health and safety regulations. Ghana, currently has no occupational health and safety laws. The lack of these sector related regulations contribute massively to the morbidities and mortalities the sector records. In this instance, there is emphasis on self-care, where the onus is on the miners to protect themselves at work. To develop and increase miners health care awareness and perception, the complexities in the sector with regards to occupational health has to be disentangled for better understanding.

The findings of this study showed that the subsector and department of goldminers play an important role in the occurrence and experience of work related health problems. Here, subsector department was robust and persisted in predicting the likelihood of experiencing occupational related health problems in all three models. Goldminers who were in ASM non-production, LSM production and LSM non-production were less likely to experience occupational related health problems as compared to their counterparts in ASM production. This is a clear indication that goldminers in the LSM subsector have a very smaller chance of experiencing occupational related health problems as compared to their counterparts in ASM. This also means that subsector and department differences in exposure to risk factors as well as varying social situations produce subsector and department specific patterns of occupational health problems. This finding is supported by [11] and [12] who stated that experiencing work related health challenges depends on the subsector of the miner.

Despite the non-existence of occupational health and safety laws in Ghana, the LSM subsector by virtue of association is guarded by several international health and safety standards and regulations. LSMs are big multinationals that run huge operations all over the world, hence their health and safety scope goes beyond Ghana. The LSM is characterized by increasing levels of investments which is improving the health conditions of its workers. They are modernized with the latest mining technology and heavy equipment therefore reducing the dangers of excessive manual work which usually results in accidents. The introduction of mechanical ventilation systems, water fed drills and restrictions on blasting are a few of the efforts by the LSM to make the mine safe and healthy. Some LSMs have comprehensive health care for their workers and own their hospitals or engage the services of private hospitals to provide quality health care for their workers. Groves et al. [13] reported in their study that off-road ore haulage was the most common source of fatalities in the LSM sector.

ASM on the other hand offers a direct, autonomous and rapid prospect of earning income to mostly indigenous people. It is publicly known that ASM miners operate in unsafe conditions which poses serious threats to their health. The reliance on elementary methods in the subsector results in high number of occupational diseases and accidents. Most ASMs operate in rural areas aligning themselves to local customs and usually outside the bounds of Ghana’s legal framework. This contributes to its lack of compliance mechanisms and well established operating systems. It is not uncommon for ASM workers to experience bodily injuries from rock falls, faulty machinery and collapse of tunnels. Although, the Personal Protective Equipment at Work Regulations 1992, require employers to provide their employees with suitable PPEs, the employers in ASM often ignore the regulatory requirement [14]. ASM miners generally operate without PPEs. Some of the ASM miners interviewed in this study operated without the use of PPEs. Miners who had safety boots obtained them as gifts from friends who worked in LSMs. Most ASM miners preferred rubber sandals which they believed provided firmer grip on the ground and prevented them from falling. Other miners who worked around crushers were seen using rugs as nose masks instead of the required standard nose masks. Some interviewed miners who had experienced work related occupational health problems opined that it could have been prevented, with many citing personal protective equipment as a solution.

The findings also showed that the department of the miners also determined the likelihood of experiencing work related health problems. In this instance, ASM miners in production departments were more likely to experience work related health problems. This is supported by [15] who stated that the activity of the miner significantly influenced occurrence of occupational health problems. Activities in the production departments such as excavation, crushing, drilling, working underground are well known areas and sources of health hazards in the ASM subsector.

Of the compositional factors, gender, marital status and age influenced the goldminers’ chances of experiencing work related health challenges. Females in this case were more likely to experience occupational related health challenges as compared to their male counterparts. This finding is in tandem with [15] who posited that injury rate in goldmining were higher for women than men. Mining worldwide has been a male dominated occupation due to the nature of the work. In less developed countries where the mine is less automated and sometimes disorganized, the physicality and brute force required for many operations might expose females, classified as vulnerable, to occupational health problems. There is also a high level of discrimination in the mining industry, especially in ASM. Male and female miners have unequal access to resources (financial and protective gears). Dinye and Erdiaw-Kwasie [16] posited in their study in the Tarkwa mining area that men were given more PPEs for self-protection than their female counterparts. This meant that the company’s health and safety policies favored men hence discriminating against women. Also, goldminers who were married in this study were more likely to experience occupational related health problems. This could be as a result of their extra household responsibilities predisposing them to health hazards at work.

The findings also showed that older miners were more likely to experience occupational related health problems as compared to the relatively younger counterparts. This is backed by [17] who reported that age of employees influence their experience of occupational health issues. Age to an extent is a function of experience. The older miners in this study could have been predisposed to occupational diseases because of their longer years in the sector hence experiencing occupational related health problems.

Among the contextual factors, goldminers who worked on shift based regimes were more likely to experience occupational related health problems. Horwitz and McCall [18] stated in their study that shifts regimes, particularly night shift, increased the risk of injury among workers. This could mean miners who work on shift regimes, especially in the nigh, are exposed to more health hazards as compared to miners who work in the day. Additionally, shift-based schedule that alternate between daytime and night can leave miners at risk of occupational fatigue. This increases miners’ chances of experiencing occupational related injuries. Also in this study, miners who lived closer to mine sites were more likely to experience occupational related health challenges. The distance between mining sites and places where the public reside is to some extent regarded as a natural barrier to direct transmission of diseases from the mining areas to the distant living areas of people. Accidents and diseases in the vicinity around the mining areas cannot be completely separated from mining activities in the mining area. Some accidents and diseases outside the mining area might also be attributed to mining activities. For example, problems emerge when mining products are hauled to different places. There are reports of increase in acute respiratory infections in this case. People who live in close proximity to mining operations, especially ASM, see the mining activities as major source of nuisance which could endanger their health.

Of the working conditions, health and safety conditions were significant predictors of experiencing occupational related health challenges. Goldminers who reported good health conditions at the work place were less likely to experience occupational related health problems as compared to those who reported poor health conditions. Scott and Grayson [17] posits that working conditions affect miners’ experience of occupational related health problems. Working in good health conditions should normally translate into better health outcomes. Counterintuitively, goldminers who reported good safety conditions had higher chances of experiencing occupational related health challenges as compared to those who reported poor safety conditions. By virtue of repetitive exposure, goldminers who experience occupational related health problems could perceive risks to be low or have a false sense of security in rating the safety conditions at the workplace when indeed they work under very poor safety conditions. Didla [19] posits that if workers perceive their workplace risks to be low, they are less likely to have a good health and safety culture. It is however not surprising that miners who indicated they work in good safety conditions still had a higher chance of experiencing occupational related health challenges.

Overall, the subsector department of miners affected their likelihood of experiencing occupational related health problems. Although compositional attributes, contextual factors and working conditions had significant effects on the likelihood of experiencing occupational related health problems, it had very minimal influence on the effect of subsector department on experiencing occupational related health problems. The study showed a distinct relationship between subsector and department of a goldminer and experiencing occupational related health challenges in Ghana. It is recommended that in managing occupational health at work, the different scenarios in exposure to health hazards between the subsectors and departments should be examined and managed. Programs that increases the awareness of risk and a good self-care culture should be promoted especially in the ASM subsector. PPE’s could be relied on for protection in the mining industry but they should be the last line of defense and not to replace preventive measures.

### Study limitations

The use of self-reported measures is a limitation in this study. There is the likelihood of miners disgruntled about issues unrelated to the theme of this study to report distorted responses. To neutralise this limitation, participants were assured of their anonymity and promised that their responses will be treated with a high level of confidentiality. It is well documented that self-reported measures have proven to be effective for organizational health and safety studies [11,20–24]. Notwithstanding the limitation of using self-reported measures, our findings showed a clear relationship between subsector department and the likelihood of experiencing occupational related health problems.

## Conclusion

In managing occupational health problems, a simple comprehension of the main determinants of the condition is required. The independent effect of the subsector department, compositional attributes, contextual factors and working conditions on goldminers’ likelihood of experiencing occupational related health challenges was evaluated using a nested binary logistic regression model. Subsector department was robust and persisted in predicting experiencing occupational related health problems in all three models. Goldminers who were in ASM non-production, LSM production and LSM non-production were less likely to experience occupational related health problems as compared to their counterparts in ASM production. For compositional factors, female goldminers were more likely to experience occupational related health problems as compared to their male counterparts. Miners who were married and also older miners were more likely to experience occupational related health problems as compared to the unmarried and the relatively younger goldminers. At the contextual level, miners who worked on shift-based schedule and also miners who lived close to mine sites were more likely to experience occupational related health problems. Among the working conditions, goldminers who worked in good health conditions were less likely to experience occupational related health problems. Counterintuitively, goldminers who rated their safety conditions as good were more likely to experience occupational related health problems. Gold miners are exposed to different health risk scenarios across subsectors and departments. These conditions need critical attention and action from industry stakeholders. Programs that increase the awareness of risk and a good self-care culture should be promoted especially in the ASM subsector. PPE’s could be relied on for protection in the mining industry but they should be the last line of defense and not to replace preventive measures and actions.
